# The Perspectives of Community Pharmacists Toward the Name-Based Rationing System During the COVID-19 Pandemic in Taiwan: Cross-Sectional Survey Study

**DOI:** 10.2196/60000

**Published:** 2024-10-24

**Authors:** En-ling Chen, Chyi-Huey Bai, Paul T Kocis, Wenke Hwang

**Affiliations:** 1 Department of Clinical Pharmacy College of Pharmacy Taipei Medical University Taipei Taiwan; 2 Department of Public Health Sciences Penn State College of Medicine Hershey, PA United States; 3 Department of Public Health College of Medicine Taipei Medical University Taipei Taiwan; 4 Department of Pharmacology Penn State College of Medicine Hershey, PA United States

**Keywords:** name-based rationing system, NBRS, community pharmacy, community pharmacist, COVID-19, SARS-CoV-2, KAP, knowledge, attitude, and practices, public health, health emergencies, government strategy, mobile phone

## Abstract

**Background:**

In Taiwan’s public health system, community-based pharmacists are key first-line health care providers due to their high accessibility. During the COVID-19 pandemic, the pharmacists played a central role in the distribution of these supplies through the Name-Based Rationing System (NBRS), during an acute shortage of masks and testing kits, that helped reduce the spread of the disease. The NBRS, an innovative government-guided strategy developed after the COVID-19 outbreak, provided equitable and convenient access to masks and COVID-19 test kits.

**Objective:**

This study aimed to investigate (1) Taiwanese pharmacists’ knowledge, attitude, and practices (KAPs) of COVID-19, with the intention to assess their preparedness for public health emergencies and their capabilities to implement COVID-19–related policies effectively; (2) their perspectives toward the NBRS; and (3) the association between individual’s KAP and corresponding perspectives toward the NBRS.

**Methods:**

A cross-sectional, web-based survey was conducted in 2 major cities in Taiwan, from June 18 to September 11, 2022, during the peak of the COVID-19 pandemic. To gauge community pharmacists’ KAP, a 66-question instrument was developed using guidelines from the Taiwanese Centers for Disease Control, the International Pharmaceutical Federation, and the Taiwanese Pharmacist Association. The instrument’s internal consistency reliability was ascertained using Cronbach α (0.819), and its content validity was verified by field experts.

**Results:**

Overall, 343 Taiwanese community pharmacists were recruited in the study. Among them, 88% (303/343) scored high on knowledge domain questions related to SARS-CoV-2; 58% (201/343) and 39% (136/343) held positive and neutral attitudes toward COVID-19–related policies, respectively; and 77% (266/343) practiced infectious disease prevention measures in compliance with official guidelines. The results demonstrated a high level of competency in pharmacists in a public health crisis. It revealed that factors including age, pharmacy characteristics, and the number of customers were associated with their perceptions and willingness to continuously participate in the NBRS. Overall, the community pharmacists showed greater support for the COVID-19–testing NBRS compared with the mask NBRS, because of the more favorable influence on the revenue and workforce of the pharmacies and the well-being of the pharmacists. Responses also highlighted concerns about rapid government policy changes and supply dynamics, underscoring the importance of effective communication and considering supply availability in facilitating a successful NBRS.

**Conclusions:**

The strong KAP of the community pharmacists justified the government leveraging their expertise in Taiwan’s COVID-19 response. While community pharmacies have proven to be essential distribution centers through the NBRS, improving community connections, communication with the government, and supply management are recommended to strengthen the system. These potential approaches aim to ensure successful NBRS implementation and better preparedness for future public health emergencies. Overall, pharmacists have demonstrated their integral role in achieving equitable outcomes and their dedication to public health efforts during crises.

## Introduction

### Background

COVID-19 infection, a highly infectious respiratory disease caused by the novel SARS-CoV-2, has significantly impacted global health care systems [1,2]. Despite Taiwan’s geographic proximity to the initial epicenter of the COVID-19 outbreak, its swift response and containment policies during the early stages have allowed Taiwan to maintain a relatively controlled situation compared with other countries [3-5]. The first surge of COVID-19 cases in Taiwan occurred in late April 2021, with a total of 8924 and 4871 confirmed cases in May and June 2021, respectively [3]. This facilitated the government to implement stricter nationwide control measures, such as a mask mandate, a ban on in-restaurant dining, work-from-home policies for nonessential businesses, and the cancellation of social and religious gatherings, resulting in Taiwan reporting zero daily cases by October 2021 [6,7]. Amid the ongoing efforts to combat the COVID-19 pandemic, community pharmacists leveraged their existing network with the public and ensured access to essential supplies and health care services to support communities, making them valuable assets in times of crisis.

In Taiwan, community-based pharmacists are often viewed as the first-line health care providers in the public health system [8]. During the COVID-19 pandemic, the high visibility and accessibility of community pharmacies made them key hubs for providing necessary health services, including medication, preventive supplies, and consultation [9]. In addition to the standard services provided by pharmacies, there were several expanded roles of community pharmacists introduced after the COVID-19 pandemic outbreak, such as home delivery, telehealth consulting, and serving as the distribution center of the prevention supplies, bringing great convenience to the public [9,10].

### Name-Based Rationing System

In response to the COVID-19 outbreak, the Taiwanese government implemented the Name-Based Rationing System (NBRS) to ensure that the public had access to adequate infection prevention supplies (eg, masks and COVID-19 testing kits) [6,11]. This system relied on the Taiwanese community pharmacies to help distribute supplies while preventing chaotic purchasing behavior and public panic during the global crisis [3,8,12,13]. Furthermore, this safeguarded the health care system by prioritizing the needs of the health care providers for masks during distribution [3,14]. An important feature of the NBRS was its use of the National Health Insurance (NHI) system. The NHI, a government-managed health insurance program covering about 99% of the Taiwanese population [15,16], recognized the ID numbers of the individuals once they received health care services from NHI-contracted health care institutions, including community pharmacies. Consequently, the NHI data collection system received uploads of their electronic health records, encompassing diagnoses, procedures, and medications, and is now equipped with records of supply purchases connected to the NBRS [14].

Under the NBRS, individuals could purchase preventive supplies on designated days based on the last digit of their NHI card number. For instance, those with an odd-numbered NHI card could purchase masks or test kits on Mondays, Wednesdays, and Fridays, while those with even-numbered cards could do so on Tuesdays, Thursdays, and Saturdays. Sundays were open to all to prevent stockpiling [14]. Pharmacists could check the eligibility of the customers and whether the masks or testing kits were repeatedly purchased [14]. Therefore, the term “Name-Based” in the NBRS originates from its reliance on individuals’ NHI cards for health care services and records, ensuring transparency and preventing exploitation of essential health supplies during a public health emergency.

Moreover, a survey of Taiwanese residents reported that 95.5% of participants believed mask wearing was protective of COVID-19 infection [17]. The study also indicated a high level of satisfaction from the public, as the system provided equitable distribution of supplies and pre-emptied possible price gouging among distributors, effectively alleviating overall anxiety [17].

### Challenges

Despite the benefits of the NBRS to ensure fair distribution of medical supplies, pharmacists faced several challenges while implementing the system. One major challenge was managing the large numbers of the public requesting supplies, which created long queues of people waiting and at the same time increased the risk of COVID-19 transmission [12]. Another significant challenge faced by pharmacists was the time needed to individually package the masks that were initially provided in bulk into small units [8]. Unlike several other countries where the role of pharmacy technicians is well established, Taiwan lacks this support system. As a result, the newly developed NBRS collaborating with the community pharmacy may result in burdensome time shifts for pharmacists, impacting their ability to carry out medication dispensing, patient education, and other essential services.

While several studies have discussed the advantages of the system [6,12,18,19], there is a lack of research on the perspectives of the NBRS practitioners, hindering a comprehensive understanding of the potential impacts of this system on community pharmacies. Despite the government’s selection of community pharmacists to implement the NBRS, likely due to the high accessibility, existing community connections, and clinical expertise, a gap exists in assessing their preparedness for public health emergencies and their necessary capabilities to implement COVID-19–related policies effectively. This study seeks to address this by investigating Taiwanese community pharmacists’ knowledge, attitudes, and practices (KAP) toward COVID-19, as they play important roles in controlling the pandemic by disseminating accurate information, educating customers on preventive measures, and serving as the distribution center of essential supplies, which reached a broader population within the communities.

Therefore, the study aimed to fill the aforementioned gap in the literature by examining (1) pharmacists’ KAP, (2) their perspectives toward the NBRS, and (3) the association between an individual’s KAP and corresponding perspective toward the NBRS. By exploring these research questions, we can gain a more comprehensive understanding of the factors influencing the perspectives of the pharmacists toward public health interventions during pandemics, and better support pharmacists in their important roles in the next global health crisis.

## Methods

### Study Design

A cross-sectional, web-based questionnaire was distributed in Taiwan from June 18 to September 11, 2022, during the COVID-19 pandemic, to analyze pharmacists’ KAPs of COVID-19 and the impact of the NBRS on community pharmacies.

### Questionnaire Development and Structure

The questionnaire was developed and referenced from multiple sources, including the International Pharmaceutical Federation, the official website of the Taiwanese Centers for Disease Control, the pandemic-preventive guidelines for community pharmacies issued by the Taiwanese Pharmacist Association, and previous KAPs or pandemic preparedness survey research conducted among pharmacists in other countries [20-30]. We assessed the internal consistency reliability of the questionnaire using Cronbach α, which demonstrated strong internal consistency across different sections: KAP survey (Cronbach α=0.819), NBRS mask section (Cronbach α=0.774), and NBRS COVID-19–testing section (Cronbach α=0.838). The content validity was ascertained with a team of 2 pharmacists, 2 epidemiologists, and a survey research consultant. This team carefully reviewed the questionnaire and provided feedback on the appropriateness and relevance of the questions to the covered construct. Based on their feedback, necessary revisions were made to enhance the clarity of the questionnaire.

The final questionnaire comprised 66 questions divided into demographics, KAPs, and perceptions of the NBRS effectiveness. Demographics included age, sex, pharmacist ownership (pharmacy owner or employed pharmacist), years of work experience, and daily working hours. Pharmacy characteristics included location, pharmacy type (chain or independent), and the number of customers.

The subsequent section focused on pharmacists’ KAP. The knowledge measured community pharmacists’ knowledge level of COVID-19, including SARS-CoV-2’s transmission, symptoms, treatments, and preventive measures according to evidence-based information. It was designed with multiple-choice questions with right, wrong, or uncertain options about the COVID-19–related statements to measure their extent of knowledge about the disease. The attitude investigated pharmacists’ attitudes toward the effectiveness of controlled policies, vaccination, and the responsibility of health care professionals to possess and share accurate COVID-19–related information. It comprises 6 questions on a 5-point Likert scale (strongly disagree=1, disagree=2, neutral=3, agree=4, and strongly agree=5). The practices were divided into self-oriented (SO) and customer-oriented (CO) practices to evaluate pharmacists’ own disease-preventive behaviors and their implementation of advising customers to adhere to the guidelines. SO practice involved actions such as handwashing, wearing personal protective equipment, and maintaining social distancing, whereas CO practice investigated whether the pharmacists prompted customers to wear masks, measure temperature, sanitize before entering the pharmacy, and other preventive behaviors. In total, 16 questions on a 5-point Likert scale according to the frequency of practicing preventive measures were included in the practice section, with never=1, rarely=2, sometimes=3, often=4, and always=5.

Finally, the questionnaire queried pharmacists’ perceptions of the NBRS for masks and COVID-19 testing to evaluate the impact on revenue, workforce, and pharmacists’ well-being. Furthermore, the questionnaire encompassed open-ended questions for both mask and COVID-19–testing systems concerning additional consequential impacts of the NBRS on pharmacists or their affiliated pharmacies.

### Data Collection

A convenience sampling method was used for data collection in 2 major cities in Taiwan (Taipei City and New Taipei City), which were also the cities with the highest COVID-19–caused deaths [3,18] and the largest impact of the NBRS on the community [11]. The total number of the contracted pharmacy in these regions was 1923. We calculated the target sample size (n=321) with the formula below to generalize pharmacies in Taipei City and New Taipei City with a 5% margin of error and 95% CI.



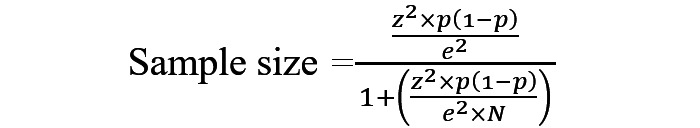



**Figure 1:**
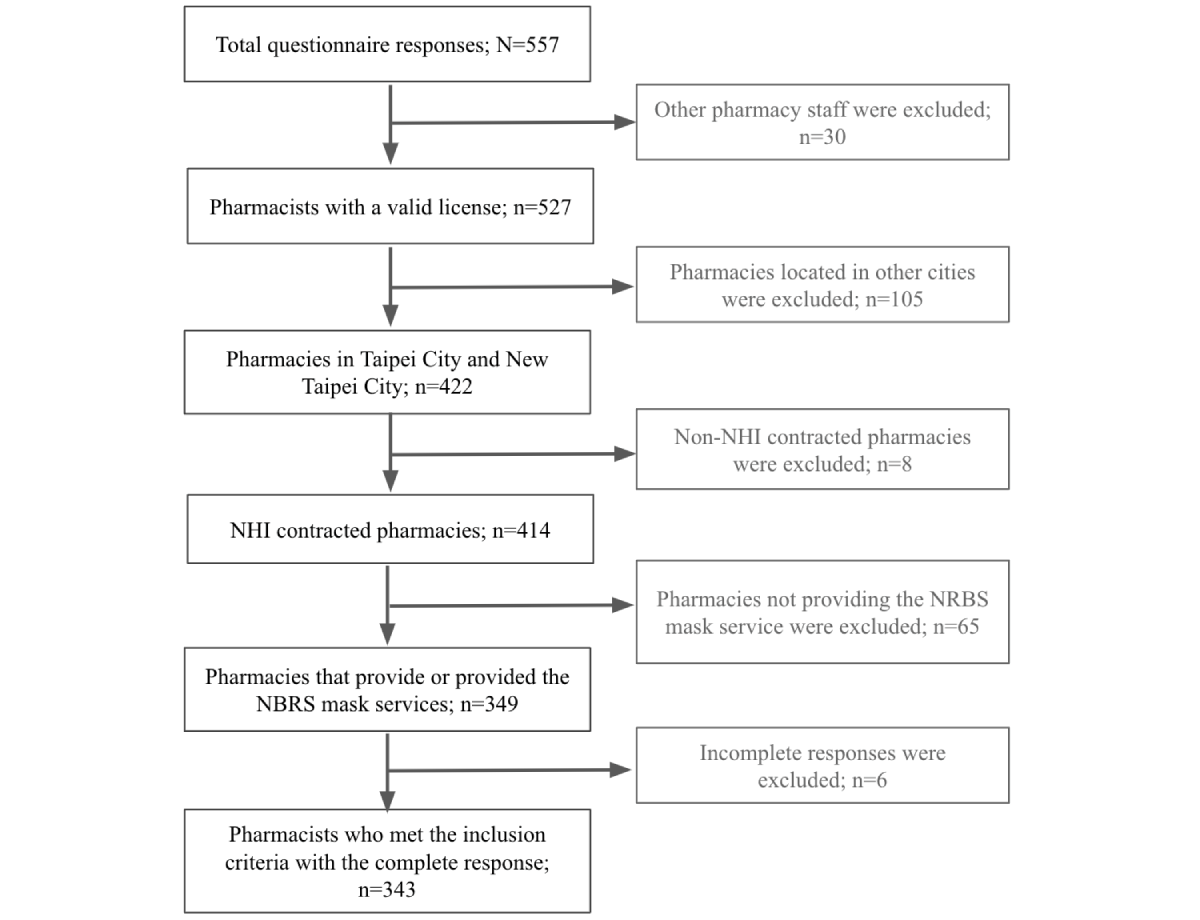
Inclusion criteria flowchart of the cross-sectional, web-based questionnaire for community pharmacists and their affiliated pharmacies in 2 major cities (Taipei City and New Taipei City) in Taiwan (N=557). NBRS: Name-Based Rationing System; NHI: National Health Insurance.

This web-based survey was distributed through social media platforms (eg, Facebook [Meta] and LINE [Line Corporation]). The cover page of the questionnaire included a short introduction of the study objectives, inclusion criteria, declarations of anonymity and confidentiality, and the voluntary nature of participation. The inclusion criteria for the subjects were determined as follows, with the accompanying flow chart visually representing the process (Figure 1). Participants failing to meet the predetermined inclusion criteria were automatically excluded through the questionnaire settings. Upon application of the inclusion and exclusion criteria, incomplete responses were excluded (n=6), with only fully completed responses (n=343) being included in the final analysis. The criteria included (1) full-time or part-time community pharmacists with a valid pharmacist license; (2) pharmacists that work in NHI-contracted pharmacies located in Taipei City and New Taipei City; and (3) pharmacists that were or are currently responsible for the named-based rationing of masks or testing.

### Statistical Analysis

The data from the questionnaire were exported into Microsoft Excel and IBM SPSS Statistics (version 18). Descriptive analyses were measured as frequency and percentage for categorical variables and mean (SD) for continuous variables. Scores of questions on knowledge (8 questions, range of score 0-8), attitudes (6 questions, range of score 6-30), and practices (15 questions, range of score 15-75) were computed by adding the score of each item. The knowledge section initially featured 10 questions. A total of 2 questions were subsequently omitted: one due to the swift evolution of COVID-19 treatment and another following Taiwan’s quarantine policy change. In this knowledge section, 1 point was assigned to the correct answer and 0 to the wrong and uncertain answer. Questions for attitude and practices using a 5-point Likert scale were added up for further analysis. We used Bloom cutoff points to classify scores into categories of high, moderate, and low, following established methodology in KAP studies [30-32]. Specifically, scores between 80% and 100% were considered high, between 60% and 79% as moderate, and below 60% as low. For the practice sections, divided into SO and CO practices, we used modified cutoff points based on the distribution of scores within our sample to create meaningful groupings. The cutoff points for high, moderate, and low were ≥7, 5-6, ≤4 for knowledge; ≥25, 19-24, ≤18 for attitudes; ≥36, 26-35, ≤25 for SO practice; and ≥21, 11-20, ≤10 for CO practice. Pearson correlation coefficient and multilinear regression analyses were conducted to examine the relationship between KAPs concerning COVID-19 prevention among pharmacists.

Furthermore, the assessment of pharmacists’ perceptions of NBRS effectiveness was calculated by combining the positive effect score and the reversed negative effect score, resulting in a total range of 10-60. The mean difference of the NBRS effect score among different sociodemographic characteristics was compared using the independent sample 2-tailed *t* test and one-way ANOVA. The web-based survey was anonymous as per the study protocol; thus, we have no way to ascertain which region the survey participants were from. However, we inspected the data distribution for the responses from each question and ascertained that the data were relatively normally distributed. Pearson correlation coefficients were calculated and tested. Univariate and multivariate linear regression analyses were used to determine the significant variables within sociodemographic characteristics, pharmacy characteristics, and KAP that affect the perspectives of the pharmacists on NBRS effectiveness. The related coefficient, SE, and 95% CI values are shown. *P*<.05 indicated that the 2-sided *P* value was significant.

### Ethical Considerations

This study was reviewed and approved by the expedited review process of the Taipei Medical University-Joint Institutional Review Board (N202205055). It was subsequently reviewed by the Human Subjects Protection Office of Penn State University, which determined that the proposed activity—secondary analysis of deidentified data—did not meet the definition of human subject research and that institutional review board review and approval were not required (STUDY00021659). The informed consent descriptions were stated at the beginning of the questionnaire, including a declaration that the survey was solely for research purposes, along with the study objectives, inclusion criteria, and declarations of confidentiality. On the same declaration page, participants were informed about the voluntary nature of their participation, with a statement that proceeding to provide responses to the survey indicated their consent to participate. To protect privacy and confidentiality, all data were deidentified before analysis. Participants were compensated with a coupon amounting to NT $100 (approximately US $3), as approved by the institutional review board. This paper contains no photos that identify individual participants.

## Results

### Pharmacists’ Demographics

Among 343 Taiwanese community pharmacists, 53.6% (184/343) were male and 46.4% (159/343) were female. As for the pharmacist age distribution, the most common age groups were 31-40 years (101/343, 29.4%) and 21-30 years (89/343, 25.9). In addition, the majority (242/343, 70.6%) were employed pharmacists, while 32.4% (111/343) were pharmacy owners. In terms of the region of the pharmacy, 48.1% (165/343) were located in New Taipei City and 51.9% (178/343) in Taipei City. The participants’ sociodemographic information and pharmacy characteristics are shown in Table 1.

**Table 1:** Demographic and professional experience of community pharmacists (n=343).

Categories and their groups		Community pharmacists (n=343), n (%)
**Sex**		
	Male	184 (53.6)
	Female	159 (46.4)
**Age group (years)**		
	≤30	89 (25.9)
	31-40	101 (29.4)
	41-50	75 (21.9)
	51-60	44 (12.8)
	Over 60	34 (9.9)
**Pharmacy ownership**		
	Pharmacy owner	111 (32.4)
	Employed	232 (67.6)
**Work hours per day**		
	<5	18 (5.2)
	5-8	139 (40.5)
	>8	186 (54.2)
**Pharmacy location**		
	Taipei City	178 (51.9)
	New Taipei City	165 (48.1)
**Pharmacy characteristics**		
	Independent pharmacy	242 (70.6)
	Chain pharmacy	101 (29.4)
**Number of customers per day**		
	<50	47 (13.7)
	51-100	136 (39.7)
	101-150	96 (28.0)
	151-200	33 (9.6)
	201-250	14 (4.1)
	>250	17 (4.9)

### KAP of Taiwanese Pharmacists Regarding COVID-19

Research findings indicated that among 343 pharmacists, the mean knowledge score for COVID-19 was 7.22 (SD 0.73; range 3-8). Scores of ≥7 were categorized as high, 5-6 as moderate, and scores of ≤4 as low, with 88.3% (303/343) demonstrating good knowledge levels and 11.3% (39/343) showing moderate levels. The mean score for attitude questions was 25.33 (SD 2.97; range 10-30). The cutoff points for attitude score were ≤18 as low, 19-24 as moderate, and ≥25 as high. The majority of participants had a positive (58.6%, 201/343) or neutral (39.7%, 136/343) attitude toward the controlled policies, vaccination, and the responsibility of health care professionals. A total of 77% (266/343) of the participants demonstrated good compliance with the guidelines in SO practice behaviors (75.2%, 258/343) and CO practice behaviors (78.7%, 270/343), with the cutoff points ≤25 (low), 26-35 (moderate), and ≥36 (high) for SO practice and ≤10 (low), 11-20 (moderate), and ≥21 (high) for CO practice (Table 2). The findings indicated that pharmacists exhibited high professional competency and individual responsibility in implementing COVID-19–preventive measures.

**Figure 2:**
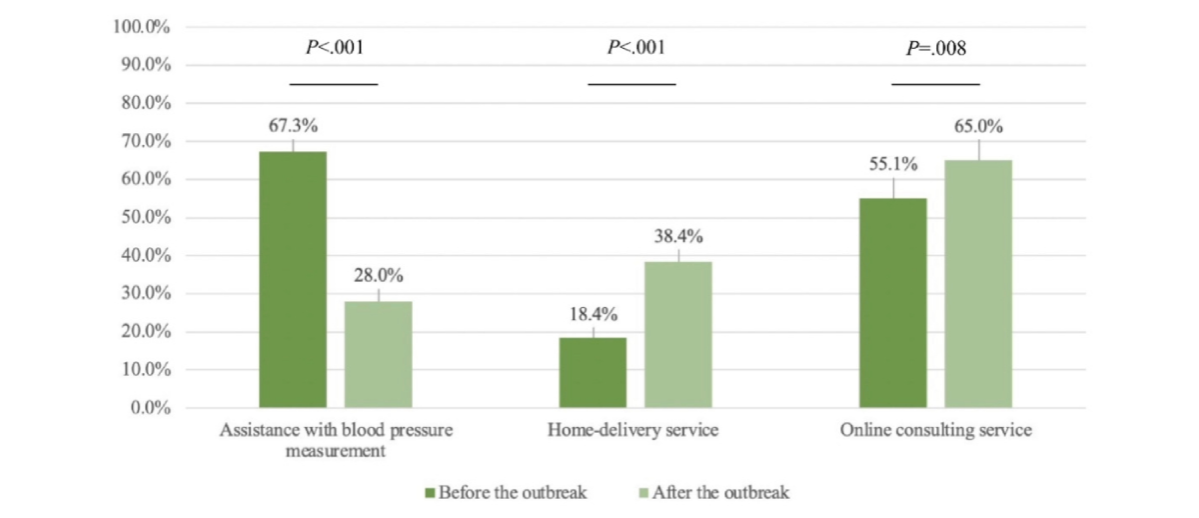
Changes in pharmacy-provided services before and after the COVID-19 outbreak.

The results also noted some changes in services during the COVID-19 pandemic among community pharmacies. In the past, assistance with measuring blood pressure was one of the common services offered in Taiwan’s community pharmacies; however, this practice was decreased due to the need for direct contact with patients that could increase the risk of spreading COVID-19. Conversely, home delivery and telehealth, 2 evidence-based strategies that were not widely provided before the outbreak, were increasingly used to reduce contact with potential or confirmed cases of COVID-19. Although there was no mandate from the Taiwanese Centers for Disease Control for these services, the community pharmacy has gone through various shifts in preventive strategies during the pandemic. Among 262 pharmacies that measured blood pressure for customers, we found a 39.3% decrease (*P*<.001) in providing this service, with 41.6% (109/262) pausing it completely and 14.1% (37/262) adjusting to letting customers measure by themselves after the pandemic outbreak. Furthermore, pharmacies that provided home delivery services significantly increased from 18.4% to 38.4% (*P*<.001) and the percentage of web-based consulting services also increased by 9.9% (*P*=.008), as shown in Figure 2.

The multivariate linear regression analysis, with practice as the dependent variable and knowledge and attitude as independent variables, revealed that attitudes significantly influenced practices related to COVID-19 prevention among pharmacists (b=0.872; *P*<.001). Specifically, the positive attitude toward preventive behaviors related to COVID-19 was strongly associated with a higher likelihood of practicing SO and CO preventive behaviors. In contrast, knowledge was not found to have a significant effect on attitudes or practices.

**Table 2:** Descriptive analysis of pharmacists’ knowledge attitude practices of COVID-19, number of questions, range of score, total score (mean, SD), and percentage of low, moderate, and high scores for each section (n=343).

Variables^a^		Questions, n	Score, range	Total score, mean (SD)	Percentage^b^ of respondents (cutoff points; n=343)		
					Low	Moderate	High
**Knowledge**		8	3-8	7.22 (0.73)	0.4 (≤4)	11.3 (5-6)	88.3 (≥7)
**Attitudes**		6	10-30	25.33 (2.97)	1.7 (≤18)	39.7 (19-24)	58.6 (≥25)
**Practices**							
	SO^c^	9	23-45	1.8 (≤25)	1.8 (≤25)	22.8 (26-35)	75.4 (≥36)
	CO^d^	6	6-30	1.7 (≤10)	1.7 (≤10)	19.6 (11-20)	78.7 (≥21)

^a^Variables: level of knowledge, attitude, and practice is determined based on the cumulative score of items within each variable.

^b^The proportion of pharmacists falling into the categories of poor, moderate, and high levels for each knowledge, attitude, and practice variable.

^c^SO: self-oriented.

^d^CO: customer-oriented.

### Relationship Between KAP Among Pharmacists

Pearson correlation coefficient tests indicated there was a positive and statistically significant correlation between the attitude and practice scores: attitude and SO practice (*r*=0.279; *P*<.001), attitude and CO practice (*r*=0.204; *P*<.001), and attitude and combined SO and CO practices (*r*=0.275; *P*<.001; Table 3). Despite measuring different behaviors, SO and CO practices were both associated with pharmacists’ attitudes, a trend that persisted when combining SO and CO practices. This suggests that engagement in one practice is likely to coincide with engagement in the other (Table 3).

**Table 3:** Correlation analysis between pharmacists’ attitudes toward COVID-19–preventive measures and their implementation of associated practices. The knowledge scores were not significantly correlated with attitudes or practices.

Attitude	Correlation coefficient	*P* value
SO^a^ practice	0.28	<.001
CO^b^ practice	0.20	<.001
Combined SO and CO practices	0.28	<.001

^a^SO: self-oriented.

^b^CO: customer-oriented.

### Impact of the NBRS

#### Overall Impact of the NBRS on Community Pharmacies

**Figure 3:**
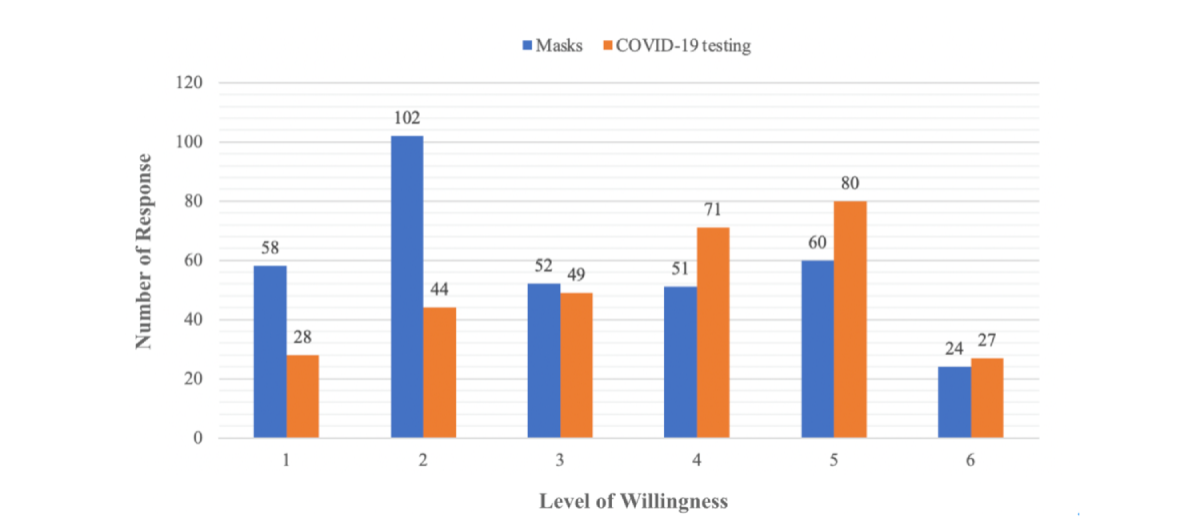
The community pharmacists’ willingness to continue implementing the mask and COVID-19 testing name-based rationing system.

To examine the overall impact of the NBRS, the survey instrument includes 3 areas of question: revenue, workforce, and pharmacists’ well-being. Data were collected separately for masks and COVID-19 testing. Overall, the community pharmacists showed greater support for the COVID-19–testing NBRS compared with the mask NBRS. This greater favorability is reflected in the willingness of pharmacists to continue participation in the NBRS in the future. Specifically, 59.7% (178/298) of responding pharmacists expressed a willingness to distribute COVID-19 testing, compared with only 38.5% (132/343) who were willing to distribute masks under the NBRS (Figure 3). This difference in willingness can be attributed to the perceived higher negative impacts on revenue, workforce, and pharmacists’ well-being for the mask NBRS compared with the COVID-19–testing NBRS (*P*<.001 for all comparisons).

In Figure 3, the x-axis represents pharmacists’ willingness to continue implementing the NBRS, graded on a scale from 1 to 6, where 6 denotes the strongest agreement with collaborating with the government to implement the system, and 1 indicates the least willingness. The y-axis corresponds to the number of responses received from pharmacists. In the chart, the mask system responses are depicted in blue, while responses related to the COVID-19–testing system are represented in orange.

#### Factors Associated with Various Impacts of the NBRS on Community Pharmacies

The study revealed various pharmacists’ perceptions of the NBRS across different demographic categories through ANOVA. Pharmacy owners exhibited higher satisfaction levels with the mask NBRS compared with employed pharmacists (*P*=.01). Age also emerged as a significant factor, with pharmacists aged 60 years and older exhibiting more positive perceptions than younger age groups (*P*<.001). Specifically, the post hoc analysis highlighted pharmacists aged years and older rated the mask NBRS higher than those aged ≤30 (*P*=.003), 31-40 (*P*<.001), and 41-50 (*P*=.006) years, but not 51-60 years (*P*=.46). As summarized, significant factors were observed in responses to the COVID-19–testing NBRS based on ownership, age, and the average number of customers.

Univariate and multivariate regression analyses were conducted while controlling for potential confounders. Table 4 shows that older pharmacists (>60 years of age; coefficient of slope; b=6.579, *P*<.001) showed a significantly positive perception for both the mask and COVID-19–testing NBRS. However, pharmacists who served more customers per day (b= –8.211, *P*<.001) showed significant negative effects on their perspective on the NBRS.

Furthermore, our study combined mask and testing scores as an indicator of pharmacists’ overall satisfaction with the NBRS. The results revealed that among their KAP scores, their attitude toward preventive guidelines, disease control authorities, and pharmacist’s added responsibilities during the pandemic significantly affected their perceptions of the NBRS, regardless of masks or COVID-19 testing (Table 4). We assessed multicollinearity in the multivariable linear regression models, finding all variance inflation factors to be below 10, indicating an acceptable level of multicollinearity.

**Table 4:** Multivariate regression analyses identifying factors influencing pharmacists’ perspectives toward the mask Name-Based Rationing System (NBRS), COVID-19–testing NBRS, and overall NBRS implementation.

Variables^a^		NBRS for mask			NBRS for testing				Overall NBRS (masks and testing)		
		Coefficient (SE)	95% CI	*P* value	Coefficient (SE)	95% CI	*P* value	Coefficient (SE)		95% CI	*P* value
**Sex (male)**		–0.13 (0.48)	–1.07 to 0.82	.79	0.35 (0.54)	–0.72 to 1.42	.52	0.36 (0.96)		–1.53 to 2.26	.71
**Age groups (years; reference=<30)**											
	31-40	–0.75 (0.63)	–1.98 to 0.49	.24	–0.02 (0.71)	–1.41 to 1.38	.98	–0.71 (1.25)		–3.18 to 1.75	.57
	41-50	–0.08 (0.67)	–1.40 to 1.25	.91	0.69 (0.76)	–0.80 to 2.19	.36	0.96 (1.34)		–1.67 to 3.6	.47
	51-60	1.14 (0.79)	–0.42 to 2.70	.15	2.08 (0.95)	0.21 to 3.95	.03	3.95 (1.68)		0.65 to 7.25	.02
	61 years and older	3.40 (0.87)	1.68 to 5.10	<.001	3.03 (0.98)	1.09 to 4.96	.002	6.58 (1.73)		3.17 to 9.99	<.001
**Ownership (reference=pharmacy owner)**		–0.70 (0.62)	–1.92 to 0.51	.26	–1.40 (0.71)	–2.80 to 0.003	.05	–1.81 (1.26)		–4.30 to 0.68	.15
**Work hours per day (reference=≤5 h)**											
	5-8 h	–0.07 (1.10)	–2.24 to 2.10	.95	–0.53 (1.22)	–2.94 to 1.87	.66	0.13 (2.17)		–4.14 to 4.41	.95
	>8 h	–0.53 (1.13)	–2.75 to 1.69	.64	–0.74 (1.25)	–3.20 to 1.72	.56	–0.72 (2.22)		–5.09 to 3.65	.75
**Location (reference=Taipei City)**		0.65 (0.47)	–0.28 to 1.58	.17	0.38 (0.53)	–0.67 to 1.42	.48	0.88 (0.94)		–0.97 to 2.73	.35
**Characteristics (reference=independent pharmacy)**		1.75 (0.59)	0.59 to 2.92	.003	1.56 (0.66)	0.26 to 2.85	.02	3.04 (1.17)		0.74 to 5.33	.01
**Number of customers per day (reference=≤50)**											
	51-100	–1.02 (0.75)	–2.50 to 0.46	.18	–2.46 (0.88)	–4.19 to –0.73	.005	–4.64 (1.56)		–7.71 to –1.57	.003
	101-150	–1.13 (0.81)	–2.72 to 0.47	.17	–1.95 (0.94)	–3.79 to –0.10	.04	–4.38 (1.67)		–7.67 to –1.10	.009
	151-200	–0.77 (1.04)	–2.81 to 1.27	.46	–2.75 (1.21)	–5.12 to –0.38	.02	–5.07 (2.14)		–9.29 to –0.86	.02
	201-250	–1.99 (1.37)	–4.70 to 0.71	.15	–2.72 (1.53)	–5.72 to 0.28	.08	–5.85 (2.71)		–11.19 to –0.52	.03
	>250	–2.14 (1.28)	–4.66 to 0.38	.09	–4.81 (1.39)	–7.55 to –2.07	.001	–8.21 (2.47)		–13.07 to –3.35	.001
**Knowledge, attitudes, and practices of COVID-19**											
	Knowledge (COVID-19 transmission, symptoms, treatment, etc)	–0.26 (0.34)	–0.92 to 0.40	.44	–0.07 (0.37)	–0.80 to 0.66	.84	–0.33 (0.66)		–1.62 to 0.97	.62
	Attitudes (COVID-19–related policies, vaccination, pharmacists’ responsibilities, etc)	0.17 (0.09)	0.01 to 0.34	.04	0.27 (0.09)	0.09 to 0.45	.004	0.44 (0.17)		0.11 to 0.76	.009
	Practices (disease-preventive behaviors)	0.03 (0.03)	–0.03 to 0.09	.35	0.05 (0.03)	–0.02 to 0.12	.18	0.06 (0.06)		–0.06 to 0.18	.34

^a^Variables showing statistical significance include age groups (51-60 years and 60 years and older), pharmacy characteristics, the number of customers/day (>250 people) and pharmacists’ attitudes toward COVID-19–related policies, the vaccination mandate, and their responsibilities to share accurate information with the public.

#### Impact Measured based on Dimensions of Revenue, Workforce, and Pharmacists’ Well-Being

A similar percentage of pharmacists (263/343, 76.7% for masks and 232/298, 77.9% for COVID-19 testing) reported that the NBRS has resulted in increased customer flow, positively impacting revenue. However, more than half (192/343, 55.97%) of the pharmacists revealed that the busy NBRS mask-related operations had negatively affected the pharmacy’s traditional business, subsequently impacting revenue. In comparison, only 40.6% (121/298) felt the same for the NBRS COVID-19–testing system. The impact on workforce was more significant, with 90.1% (309/343) of pharmacists reporting increased workload due to NBRS operations for masks and 74.8% (223/298) experiencing the same for COVID testing, resulting in a shortage of workforce.

In order to investigate the specific dimension the demographic variables were affecting, we removed the factors that showed no significant effects on the overall NBRS outcomes, including sex, work hours, and location of pharmacy (Taipei City or New Taipei City), as shown in Multimedia Appendices 1 and 2. Results found that the pharmacists’ ownership had distinct effects on the workforce. Compared with pharmacy owners, employed pharmacists were more likely to report that the system caused the increased workload and that they experienced a more pronounced negative impact from workforce shortage. Furthermore, chain pharmacies reported a more positive impact of the NBRS on revenue compared with independent pharmacies.

We also conducted a stepwise linear regression to look into each question since there were nuances between them even within the 3D. One notable finding was that among the demographic variables, pharmacists’ KAP score demonstrated as a significant predictor of their willingness to provide the NBRS service without a government mandate (*P*<.001). This means pharmacists who practiced SO or CO preventive behaviors had more favorable perceptions of the NBRS.

#### Additional Impacts of the NBRS on Community Pharmacies

The inclusion of open-ended questions in the questionnaire was crucial to capture in-depth insights from pharmacists regarding their experiences with the NBRS (Multimedia Appendices 3 and 4). A total of 2 major themes emerged from their responses, centered around customer behaviors and government policy changes. Pharmacists highlighted behavior issues such as irritable and unruly customers waiting in queue to purchase masks and COVID-19 testing, which not only disrupt pharmacy operations but also necessitate additional staffing and time. They also described concerns regarding service challenges, including the public’s misunderstanding of purchasing rules leading to disputes.

Communication issues regarding government policy changes were another prominent theme, with pharmacists feeling uninformed and lacking advance notices about the policy changes. Concerns about the supply and demand of inventory were also raised. Some pharmacists emphasized the importance of distinguishing the impact of the mask and COVID-19–testing NBRS on pharmacies based on availability. They noted that while selling the NBRS supplies can be beneficial during times of severe shortage, it may be redundant when supplies become readily available. The decision to continue selling them was contingent on factors such as manufacturer pricing and the convenience of the public (Multimedia Appendices 3 and 4).

## Discussion

### Principal Findings

The findings of this study revealed that the majority of pharmacists demonstrated sufficient knowledge, positive or neutral attitudes, and good practices toward COVID-19, indicating their competent roles in the fight against a deadly pandemic. This showed pharmacists were well versed in COVID-19 transmission, symptoms, treatments, and preventive measures. They generally viewed the COVID-19–related policies and vaccination positively and recognized their responsibility to share accurate information. In terms of practices, pharmacists demonstrated strong personal preventive behaviors (SO practice) and were proactive in advising customers on preventive measures (CO practice). At this pivotal moment, this reaffirms the government leveraging the NBRS in cooperation with the community-based pharmacy due to its convenient locations, existing relationship with the community, and, most importantly, pharmacists’ competence in facing the challenges posed by the pandemic and contributing to the public. Furthermore, our results found that among KAP, attitudes rather than knowledge significantly influenced practices related to COVID-19 prevention among pharmacists.

Building on the KAP findings, the assessment of pharmacists’ perceptions of NBRS reflected how they played a key role as the distribution center during the pandemic and the associated impacts of this government-guided strategy. Compared with the NBRS mask distribution, a more favorable perception of NBRS testing distribution was found. Pharmacists in older age groups, pharmacy owners compared with employed pharmacists, and those who have more positive attitudes toward COVID-19–related policies reported more favorable perceptions and thus demonstrated a higher willingness to continue participating in the NBRS.

Finally, responses from the open-ended questions gathered additional impacts of the NBRS reported by pharmacists. The major themes found in our qualitative analyses highlighted concerns about rapid government policy changes and supply dynamics, underscoring the importance of effective communication between the government and pharmacists and considering evolving supply availability in implementing the NBRS.

### Interpretation of Results and Comparison with Previous Work

Regarding the good KAP of pharmacists, the findings were consistent with studies conducted in other nations, such as Pakistan [23], Vietnam [24], and Egypt [25]. Results revealed that attitudes toward COVID-19–preventive measures had a significant impact on pharmacists’ practices; however, knowledge did not show any significant relationship. This relationship was also found in the previous study conducted on the general public in Taiwan [33], showing that greater effort is needed to improve practitioners’ attitudes in order to improve their compliance with the guidelines (practices), as the study showed that the increase in knowledge was not associated with the level of compliance (practices). Interestingly, the studies conducted on community pharmacists in other countries showed that knowledge and attitude both affected their practices, which pointed out that the responses may vary based on the diverse cultures, COVID-19 situation, and policies [23,32].

As for pharmacists’ perspectives on the NBRS, the results showed that pharmacists had a more gratifying experience with the NBRS COVID-19 testing than the NBRS mask distribution. This perception was primarily influenced by the challenges associated with preparing supplies, along with heightened public anxiety. Pharmacists needed to repackage masks into smaller quantities due to rationing requirements, which imposed additional time and effort on them [8]. It is suggested to assign additional personnel to handle preparatory tasks. In contrast to certain countries where the employment of pharmacy technicians is common, such a practice remains unavailable in Taiwan. In our study, 26.8% (92/343) of pharmacists reported experiencing time shifts when there is only one pharmacist responsible for all duties, which could be a great burden if they have to maintain all the original work such as medication dispensing and education while dealing with the NBRS-related services.

In contrast, testing kits were distributed to pharmacies in prepackaged boxes, likely requiring less handling and preparation. In addition, disparities in public anxiety levels were evident during the initial implementation of the mask system in 2020, when vaccinations were unavailable [33]. However, with the subsequent initiation of the testing system in 2022, coinciding with the increased prevalence of immunization, the anxiety levels among both the public and pharmacists were alleviated [3,33]. This anxiety reduction may indirectly influence the willingness of pharmacists to assume responsibility for the system (Figure 3). Responses from pharmacists also highlighted the significance of timing in delivering NBRS services. Specifically, they noted that the NBRS is deemed necessary during periods of severe shortage for masks or testing and when the price of NBRS masks and COVID-19 testing is lower compared with supplies from other manufacturers. They emphasized that providing NBRS services is most opportune when it is convenient for the public to access supplies from pharmacies (Multimedia Appendices 3 and 4). These factors likely influence the pharmacists’ decisions regarding the continuation of such services.

Furthermore, older pharmacists (>50 years of age) in this study held more positive perspectives than younger age groups (*P*<.001), primarily due to their long-standing connections with their communities. The analysis of the open-ended questions indicated that pharmacists in the older age groups exhibited a stronger sense of community contribution and a deep sense of belonging. In contrast, younger pharmacists, who may have started their pharmacies more recently, may have weaker bonds with the public and expressed a relatively lower level of community attachment. Another factor of pharmacist ownership showed that pharmacy owners had better perceptions compared with employed pharmacists. Respondents reported that the subsidy from the government for serving the public under the NBRS was given to the unit of pharmacy; thus, some funds were used for the general improvement of the pharmacy but not for the staff pharmacists.

The findings of this study can inform future policy decisions by providing valuable insights and potential approaches, such as optimizing the preparation process, enhancing communication between officials and pharmacists, and building up the connections between newer pharmacies with the communities. Based on the responses received, it is recommended that policy makers consider implementing measures to improve communication regarding rapid policy changes. This could involve informing practitioners in advance or delivering information through designated mechanisms for pharmacists to follow, especially during periods of heightened pandemic severity or the emergence of new variants. Furthermore, policymakers may consider assigning designated groups to handle the preparatory tasks such as supply packaging. This would allow pharmacists to maximize their effectiveness by focusing their time and efforts on serving the public and ensuring optimal service delivery. Finally, given the difference in perceptions based on employment, it is suggested that giving subsidies to individual pharmacists to improve their perceptions and compliance. By addressing the underlying concerns, policy makers can foster a more favorable working environment and ultimately enhance overall service quality.

### Limitations

The limitations of this study include the relatively small sample size (n=343) limited to 2 cities, Taipei City and New Taipei City. These 2 cities were focused, as they reported the highest number of COVID-19–caused deaths and were in need of strengthened epidemic prevention measures during the pandemic [3,7]. In addition, the involvement of NHI pharmacies in these cities toward COVID-19–related policies were higher than in other cities [21]. While the findings may not be generalizable to other regions, this study provides crucial insights into the experiences of pharmacists who were directly involved in pandemic preparedness in relatively high-risk areas. Nonetheless, a broader geographic sampling is needed for future studies in Taiwan to enhance objectivity and representativeness.

Another limitation was the study’s cross-sectional design, which led to the inability to determine the causal direction between variables. It is suggested that future studies collect data longitudinally to more thoroughly assess how the NBRS influences pharmacist perspectives and practices over time. Furthermore, data were self-reported by the pharmacists, which may be subject to recall bias or social desirability bias (a type of response bias). However, all participants were asked to focus on their recent experiences and were assured anonymity and confidentiality to minimize potential biases. Therefore, the results could still serve as a reference for future policies and interventions aimed to improve public health outcomes.

### Conclusions

The study highlights the crucial role of pharmacists in the fight against the COVID-19 pandemic, with a majority demonstrating sufficient KAP and good practices toward COVID-19, justifying the government leveraging their expertise during such a pivotal moment. While community pharmacies have proven to be essential distribution centers through the NBRS, several adjustments are suggested to enhance practitioners’ perceptions of the system and ensure its successful implementation. These include building stronger connections between pharmacies and their communities, assigning additional personnel to handle preparatory tasks, establishing effective communication channels between the government and pharmacists, and considering evolving supply dynamics. Overall, pharmacists have proven to be integral to public health efforts during the pandemic, underscoring their vital role and dedication in times of crisis.

## Appendix

Multimedia Appendix 1 Impact of the NBRS mask on pharmacies' revenue and workforce and pharmacists' well-being. NBRS: Name-Based Rationing System.

Multimedia Appendix 2 Impact of the NBRS testing on pharmacies' revenue and workforce and pharmacists' well-being. NBRS: Name-Based Rationing System.

Multimedia Appendix 3 Codebook for additional impacts of the NBRS mask on community pharmacies as reported in the open-ended questions. NBRS: Name-Based Rationing System.

Multimedia Appendix 4 Codebook for additional impacts of the NBRS testing on community pharmacies as reported in open-ended questions. NBRS: Name-Based Rationing System.

## Abbreviations

CO: customer-oriented
KAP: knowledge, attitudes, and practices
NBRS: Name-Based Rationing System
NHI: National Health Insurance
SO: self-oriented

## Data Availability

The datasets generated during and/or analyzed during this study are not publicly available due to the restrictions imposed by our previous institutional review board approval. According to this approval, the data can only be accessed by the research team (principal investigator and co-principal investigator) to maintain confidentiality. However, the data are available from the corresponding author on reasonable requests for aggregated data.
