# Supplementary material for: The Perspectives of Community Pharmacists Toward the Name-Based Rationing System During the COVID-19 Pandemic in Taiwan: Cross-Sectional Survey Study
Source: JMIR Form Res. 2024 Oct 24;8:e60000. doi: 10.2196/60000 (PMC11544337; doi:10.2196/60000)
Supplement: Multimedia Appendix 1 [file formative_v8i1e60000_app1.docx]

**Multimedia Appendix 1.** Impact of the NBRS mask on revenue, manpower and pharmacists’ well-being.

|  | **Revenue** | | | **Manpower** | | | **Pharmacists’ Well-being** | | |
| --- | --- | --- | --- | --- | --- | --- | --- | --- | --- |
|  | Unstandardized  coefficient | P value | VIF | Unstandardized  coefficient | P value | VIF | Unstandardized  coefficient | P value | VIF |
| age | 0.361 | .040 | 3.709 | 0.233 | .009 | 3.709 | 0.852 | .000 | 3.709 |
| Ownership | -0.396 | .172 | 1.360 | -0.313 | .033 | 1.360 | -0.101 | .730 | 1.360 |
| Characteristics | 0.765 | .008 | 1.268 | 0.158 | .275 | 1.268 | 0.737 | .011 | 1.268 |
| Number of customers | -0.130 | .200 | 1.163 | -0.102 | .046 | 1.163 | -0.088 | .389 | 1.163 |
| Knowledge | 0.161 | .231 | 1.122 | -0.050 | .460 | 1.122 | -0.031 | .819 | 1.122 |
| Attitude | 0.124 | .003 | 1.077 | 0.009 | .681 | 1.077 | 0.091 | .031 | 1.077 |
| Practices | -0.006 | .678 | 1.300 | 0.001 | .895 | 1.300 | 0.015 | .301 | 1.300 |
