# Supplementary material for: The Perspectives of Community Pharmacists Toward the Name-Based Rationing System During the COVID-19 Pandemic in Taiwan: Cross-Sectional Survey Study
Source: JMIR Form Res. 2024 Oct 24;8:e60000. doi: 10.2196/60000 (PMC11544337; doi:10.2196/60000)
Supplement: Multimedia Appendix 2 [file formative_v8i1e60000_app2.docx]

**Multimedia Appendix 2.** Impact of the NBRS testing on revenue, manpower and pharmacists’ well-being.

|  | **Revenue** | | | **Manpower** | | | **Pharmacists’ Well-being** | | |
| --- | --- | --- | --- | --- | --- | --- | --- | --- | --- |
|  | Unstandardized  coefficient | P value | VIF | Unstandardized  coefficient | P value | VIF | Unstandardized  coefficient | P value | VIF |
| Age | 0.209 | 0.226 | 3.563 | 0.248 | 0.019 | 3.563 | 0.733 | 0.000 | 3.563 |
| Ownership | -0.355 | 0.234 | 1.446 | -0.326 | 0.074 | 1.446 | -0.824 | 0.007 | 1.446 |
| Characteristics | 0.787 | 0.006 | 1.311 | 0.286 | 0.099 | 1.311 | 0.533 | 0.066 | 1.311 |
| Number of customers | -0.345 | 0.001 | 1.178 | -0.139 | 0.023* | 1.178 | -0.109 | 0.284 | 1.178 |
| Knowledge | -0.039 | 0.764 | 1.084 | -0.014 | 0.862 | 1.084 | -0.009 | 0.946 | 1.084 |
| Attitude | 0.118 | 0.005 | 1.150 | 0.052 | 0.042* | 1.150 | 0.138 | 0.001 | 1.150 |
| Practices | 0.028 | 0.057 | 1.371 | 0.004 | 0.629 | 1.371 | 0.011 | 0.489 | 1.371 |
