# Supplementary material for: The Perspectives of Community Pharmacists Toward the Name-Based Rationing System During the COVID-19 Pandemic in Taiwan: Cross-Sectional Survey Study
Source: JMIR Form Res. 2024 Oct 24;8:e60000. doi: 10.2196/60000 (PMC11544337; doi:10.2196/60000)
Supplement: Multimedia Appendix 3 [file formative_v8i1e60000_app3.docx]

**Multimedia Appendix 3.** Codebook for additional impacts of the NBRS mask on community pharmacies as reported in open-ended questions.

| **Category** | **Code** | **Example** |
| --- | --- | --- |
| 1. Customer Behavior and Service | 1a. Trust | Increases customer trust in pharmacists, making the connections between pharmacies and communities stronger. |
|  | 1b. Behavior Issues | Facing irrational customers who come to buy masks is one of the causes of negative emotions. The behavior of these customers affects the entire pharmacy operation, requiring more staffing and time from them. |
|  | 1c. Service Challenges | The public's lack of understanding of purchasing rules leads to disputes between the public and pharmacists. |
|  | 1d. Misconceptions | Many citizens think that the odd-even ID number purchasing regulation is set by each pharmacy itself, but in reality, it is set by government agencies, causing many miscommunications. |
| 1. Pharmacy Operation | 2a. Volunteer team | After NBRS mask distribution, we established a pharmacy-exclusive volunteer team for mask packing and further assistance. |
|  | 2b. Limited Space | Unable to flexibly adjust the volume of purchases, affecting storage space, and then compressing the storage volume of other goods. |
| 1. Government Policies | 3a. Communication issues | For the new policy changes regarding the NBRS mask distribution, we only know through television/live broadcast the day before the changes. The executing end cannot get adjustment information in advance. There is no good communication platform or method. |
| 1. Health and Safety | 4a. Infection risk | Increases the risk of pharmacist infection and chaos in the mask collection order |
| 1. Supply and Demand | 5a. Timing | The impact of the NBRS mask on pharmacies must be distinguished:  1. At times when masks are severely lacking (selling the NBRS masks is beneficial to pharmacies)  2. At times when masks are not lacking (selling the NBRS masks is redundant) |
| 1. Social responsibility | 6a. Disease prevention | Selling masks has negative impacts on my life and business, but I am still willing to do it because it is beneficial for epidemic prevention. |
