# Supplementary material for: The Perspectives of Community Pharmacists Toward the Name-Based Rationing System During the COVID-19 Pandemic in Taiwan: Cross-Sectional Survey Study
Source: JMIR Form Res. 2024 Oct 24;8:e60000. doi: 10.2196/60000 (PMC11544337; doi:10.2196/60000)
Supplement: Multimedia Appendix 4 [file formative_v8i1e60000_app4.docx]

**Multimedia Appendix 4.** Codebook for additional impacts of the NBRS testing on community pharmacies as reported in open-ended questions.

| **Category** | **Code** | **Example** |
| --- | --- | --- |
| 1. Customer Behavior and Service | 1a. Behavior Issues | The NBRS testing is not as chaotic as the mask system. People are more rational, but there are still some unreasonable customers. |
|  | 1b. Service Challenges | The demand for testing by the public is not as high as imagined, and the supply-demand balance between the public and pharmacies is not that tense. Therefore, the presence or absence of the NBRS has little impact on pharmacies. |
| 1. Pharmacy Operation | 2a. Increased Visibility | Increase in the number of followers on the pharmacy's social media platforms. |
|  | 2b. Limited Space | Storage takes up space. |
|  | 2c. System setup | It takes time to go through the VPN system (if not used for a long time, it logs out automatically and requires time to log back in), and the card reader is prone to damage. |
|  |  | System setup is not completed at once, causing problems with inventory and accounting at the pharmacy end. |
| 1. Government Policies | 3a. Communication issues | Government policies change rapidly, often leaving the public informed earlier than pharmacists about relevant information. |
|  | 3b. Subsidy | The employed pharmacists are the ones who are busy, and the revenue goes to the boss. Why is there no direct subsidy for pharmacist salaries but instead a subsidy for the employer? |
| 1. Health and Safety | 4a. Infection risk | Increased risk for pharmacists due to queues. |
| 1. Supply and Demand | 5a. Timing | Whether to continue selling them depends on the manufacturer's price. Currently, we are willing to sell them because the price of real-name system rapid tests is cheaper than those from other manufacturers. If rapid test kits in the free market become cheaper than the NBRS testing in the future, we won't consider selling them. |
|  |  | Similar to the NBRS mask, most pharmacies consider the convenience of the public. When medical supplies are not difficult to obtain, the necessity of the NBRS can be reconsidered. |
